# Lycopene Improves In Vitro Development of Porcine Embryos by Reducing Oxidative Stress and Apoptosis

**DOI:** 10.3390/antiox10020230

**Published:** 2021-02-03

**Authors:** Hyo-Gu Kang, Sanghoon Lee, Pil-Soo Jeong, Min Ju Kim, Soo-Hyun Park, Ye Eun Joo, Sung Hyun Park, Bong-Seok Song, Sun-Uk Kim, Min Kyu Kim, Bo-Woong Sim

**Affiliations:** 1 Futuristic Animal Resource & Research Center, Korea Research Institute of Bioscience and Biotechnology, Chungcheongbuk-do, Cheongju 28116, Korea; kogd1887@kribb.re.kr (H.-G.K.); sodany2@kribb.re.kr (S.L.); spectrum@kribb.re.kr (P.-S.J.); jmmy05@kribb.re.kr (M.J.K.); tngusdl30@kribb.re.kr (S.-H.P.); joo9818@kribb.re.kr (Y.E.J.); ck2816@kribb.re.kr (S.H.P.); sbs6401@kribb.re.kr (B.-S.S.); sunuk@kribb.re.kr (S.-U.K.); 2 Laboratory of Animal Reproduction and Physiology, Department of Animal Science and Biotechnology, College of Agriculture and Life Science, Chungnam National University, Daejeon 34134, Korea; 3 Department of Functional Genomics, KRIBB School of Bioscience, Korea University of Science and Technology (UST), Daejeon 34113, Korea

**Keywords:** lycopene, antioxidant, reactive oxygen species, mitochondria-dependent apoptosis, pig, reproduction

## Abstract

In vitro culture (IVC) for porcine embryo development is inferior compared to in vivo development because oxidative stress can be induced by the production of excessive reactive oxygen species (ROS) under high oxygen tension in the in vitro environment. To overcome this problem, we investigated the effect of lycopene, an antioxidant carotenoid, on developmental competence and the mechanisms involved in mitochondria-dependent apoptosis pathways in porcine embryos. In vitro fertilized (IVF) embryos were cultured in IVC medium supplemented with 0, 0.02, 0.05, 0.1, or 0.2 μM lycopene. The results indicate that 0.1 μM lycopene significantly increased the rate of blastocyst formation and the total cell numbers, including trophectoderm cell numbers, on Day In terms of mitochondria-dependent apoptosis, IVF embryos treated with 0.1 μM lycopene exhibited significantly decreased levels of ROS, increased mitochondrial membrane potential, and decreased expression of cytochrome c on Days 2 and Furthermore, 0.1 μM lycopene significantly decreased the number and percentage of caspase 3-positive and apoptotic cells in Day-6 blastocysts. In addition, Day-2 embryos and Day-6 blastocysts treated with 0.1 μM lycopene showed significantly reduced mRNA expression related to antioxidant enzymes (*SOD1, SOD2, CATALASE*) and apoptosis (*BAX/BCL2L1* ratio). These results indicate that lycopene supplementation during the entire period of IVC enhanced embryonic development in pigs by regulating oxidative stress and mitochondria-dependent apoptosis.

## 1. Introduction

Pigs are used as biomedical research models for studying human diseases because their anatomy and physiology are similar to those of humans [1]. For this reason, the production of high-quality embryos is important to enhance the efficiency of transgenic pig production for biomedical research. However, the development of in vitro produced (IVP) embryos remains inferior compared to those produced in vivo because IVP embryos can be exposed to various stresses not encountered in the in vivo environment, such as light, a high oxygen content, and sudden changes in pH [2]. These stress factors raise oxidative stress levels due to the generation of reactive oxygen species (ROS) [3,4].

ROS are created from external components and byproducts of the intracellular energy metabolism pathways [5]. Under in vivo conditions, embryos are transferred into the female reproductive tract and develop from the zygote to the blastocyst stage. The environment of the female reproductive tract prevents ROS formation and ROS damage through an antioxidant system [3]. For example, catalase released from oviduct epithelial cells prevents damage to the embryos by converting or removing superoxide anions of hydrogen peroxide [6]. However, an imbalance between intracellular ROS levels and the antioxidative capacity of embryos can lead to many problems, including DNA damage, mitochondrial dysfunction, lipid peroxidation, adenosine 5′-triphosphate (ATP) depletion, apoptosis, and embryo development arrest [7]. In vitro culture (IVC) conditions can increase intracellular ROS levels and decrease the antioxidative capacity of embryos [8].

Mitochondria are important intracellular organelles responsible for energy production [9]. Previous studies have demonstrated that mitochondria perform an important role in cell death pathways [10,11]. Excessive ROS generation in embryos during IVC causes mitochondrial dysfunction [12], leading to cytochrome c release [13]. The release of cytochrome c triggers apoptosome formation and caspase-3 activation, leading to apoptosis [14]. Therefore, several exogenous antioxidants, such as carotenoids [15], vitamins [16], resveratrol [17], anthocyanin [18], and melatonin [19], have been supplemented in IVC media of porcine embryos to protect mitochondria against oxidative stress.

Lycopene, a predominant natural carotenoid in tomatoes, is a strongly antioxidative molecule that has the capacity to scavenge free radicals [20] and chemically quench singlet oxygen [21]. Lycopene has been proven to quench singlet oxygen twice as efficiently as β-carotene, and 10 times as efficiently as α-tocopherol [22]. In comparison with vitamin E, lycopene has been shown to be roughly 100 times more efficient in terms of antioxidative effects [23]. In addition, lycopene exhibits powerful antioxidative capacity not only in vitro but also in an in vivo environment, preventing damage to lipids and proteins as well as DNA oxidation [3]. However, limited information is available on the effects of lycopene on the development of in vitro fertilized pig embryos. The objective of this study was to investigate the effects of lycopene treatment during IVC on the preimplantation development of in vitro fertilized (IVF) pig embryos by evaluating embryonic developmental competence, intracellular ROS levels, mitochondrial membrane potential, expression of cytochrome c and cleaved caspase-3, and apoptosis.

## 2. Materials and Methods

### 2.1. Chemicals and Reagents

All chemicals and reagents were purchased from Sigma-Aldrich Chemical Co. (St. Louis, MO, USA) unless otherwise indicated.

### 2.2. Oocyte Collection and In Vitro Maturation (IVM)

Oocyte collection and IVM were carried out as described previously with some modifications [24]. Ovaries of pigs were obtained from a local abattoir and transferred to the laboratory within 3 h in a 0.9% (weight/volume) aqueous solution of sodium chloride containing 75 µg/mL potassium penicillin G and 50 µg/mL streptomycin sulfate at 25–30 °C. Cumulus-oocyte complexes (COCs) were recovered from ovarian follicles with a diameter of 3–6 mm by using an 18-gauge needle attached to a disposable 10-mL syringe. Oocytes with even cytoplasm and more than one layer of cumulus cells were collected and washed in maturation medium, which is tissue culture medium 199 containing 0.57 mM L-cysteine, 10 ng/mL epidermal growth factor, 10% porcine follicular fluid, 25 µM β-mercaptoethanol, 10 IU/mL pregnant mare serum gonadotropin (PMSG), and 10 IU/mL human chorionic gonadotropin (hCG). Next, the COCs were cultured in 500 µL of maturation medium in humidified air with 5% CO_2_ at 38.5 °C. After 22 h of IVM, the COCs were washed and then cultured in PMSG- and hCG-free maturation medium for an additional 20–22 h.

### 2.3. In Vitro Fertilization (IVF) and In Vitro Culture (IVC)

IVF and IVC were carried out as described previously with some modifications [25]. IVF handling medium consisted of modified Tris-buffered medium (mTBM) containing 3 mM potassium chloride, 113.1 mM sodium chloride, 7.5 mM calcium chloride dihydrate, 20 mM Tris, 11 mM glucose, 5 mM sodium pyruvate, 2.5 mM caffeine sodium benzoate, and 1 mg/mL bovine serum albumin (BSA). The COCs were denuded by gentle pipetting to remove cumulus cells in Dulbecco’s phosphate-buffered saline (DPBS; Gibco-BRL, Grand Island, NY, USA) containing 1 mg/mL hyaluronidase, 100 mg/mL penicillin G, and 75 mg/mL streptomycin sulfate. Next, selected MII oocytes were washed in IVF medium, and 10–15 oocytes were placed in 48 μL of IVF medium drops in humidified air with 5% CO_2_ at 38.5 °C. To perform insemination, ejaculated semen (supplied weekly from DARBY, Anseong, Korea) was washed with DPBS supplemented with 75 μg/mL streptomycin sulfate, 100 μg/mL penicillin G and 1 mg/mL BSA. Then, the spermatozoa were resuspended in IVF medium to a final sperm concentration of 1.5 × 10^5^ spermatozoa/mL, and mixed with 48 μL of IVF medium drops containing 10–15 oocytes. After co-incubation of oocytes with sperm for 4 h, sperm attached to oocytes were removed by gentle pipetting. Zygotes were incubated in IVF medium for an additional 2 h in humidified air with 5% CO_2_ at 38.5 °C. The zygotes were then cultured in porcine zygote medium-3 (PZM-3) with 0.1% polyvinyl alcohol (PVA) in humidified air with 5% CO_2_ at 38.5 °C for 6 days.

### 2.4. Differential Staining of Blastocysts

Differential staining of blastocysts was carried out as described previously with some modifications [26]. Blastocysts were fixed in 4% paraformaldehyde (PFA) overnight and washed three times in DPBS supplemented with 0.1% PVA (PBS/PVA) for 10 min each. To permeabilize the membranes, the fixed blastocysts were placed in DPBS supplemented with 1% Triton X-100 for 1 h at room temperature. After washing three times with PBS/PVA, to block nonspecific binding sites, blastocysts were placed in PBS/PVA containing 1% BSA (blocking solution) for 6 h at 4 °C and then transferred to DPBS supplemented with 10% normal goat serum for 1 h at room temperature. The blastocysts were then incubated at 4 °C overnight with an undiluted solution of a mouse monoclonal CDX2 antibody (Biogenex Laboratories Inc., San Ramon, CA, USA). After washing three times in blocking solution, the blastocysts were incubated at room temperature for 1 h with Alexa-Fluor-488-labeled goat anti-mouse IgG (diluted 1:200) as a secondary antibody. After washing three times in blocking solution, blastocysts were mounted on glass slides with Vectashield containing DAPI (Vector Laboratories Inc., Burlingame, CA, USA). A fluorescence microscope (Olympus, Tokyo, Japan) was used to observe DAPI-labeled or CDX2-positive cells.

### 2.5. Measurement of Intracellular ROS Levels

Measurement of intracellular ROS was carried out as described previously with some modifications [27]. To measure intracellular ROS levels, 5-(and-6)-chloromethyl-20, 70-dichlorodihydro-fluorescein diacetate, acetyl ester (CM-H_2_DCFDA; Invitrogen, Carlsbad, CA, USA) was used. Day-2 embryos and Day-6 blastocysts from each treatment group were incubated in PBS/PVA mixed with 5 μM CM-H_2_DCFDA for 30 min. After incubation, the embryos and blastocysts were washed three times in PBS/PVA and placed into 40-μL droplets of PBS/PVA. Images were captured under ultraviolet light (460 nm for ROS) using a fluorescence microscope (Leica, Wetzlar, Germany). The fluorescence intensities were analyzed using ImageJ software (version 1.47; National Institutes of Health, Bethesda, MD, USA) and normalized to those of control embryos.

### 2.6. Measurement of Mitochondrial Membrane Potential (ΔΨm)

The JC-1 staining was carried out as described previously with some modifications [28]. Day-2 embryos and Day-6 blastocysts were fixed in 4% PFA overnight at 4 °C. After washing three times in PBS/PVA, embryos and blastocysts were incubated in PBS/PVA with JC-1 (1000:1) (Cayman Chemical, Ann Arbor, MI, USA) for 30 min at 38.5 °C. The membrane potential was calculated as the ratio of red florescence, which reflects high mitochondrial membrane potential (J-aggregates), to green fluorescence, which reflects low mitochondrial membrane potential (J-monomers). Then, samples were washed in PBS/PVA three times and placed into 40-μL droplets of PBS/PVA. J-aggregates and J-monomers images were captured with a fluorescence microscope (Olympus, Tokyo, Japan). The fluorescence intensity was analyzed using the ImageJ software and normalized to that of control embryos.

### 2.7. Immunocytochemical Staining

Immunocytochemical staining was carried out as described previously with some modifications [29]. Fixed Day-2 embryos and Day-6 blastocysts were permeabilized with 1% Triton X-100 in PBS for 1 h at room temperature. Samples were incubated for 1 h at room temperature in blocking solution, then incubated overnight with primary antibodies for cytochrome c (Abcam, Cambridge, UK) and cleaved caspase-3 (Cell Signaling Technology, Danvers, MA, USA) in blocking solution, according to the manufacturers’ instruction manuals. After washing three times with blocking solution, the embryos and blastocysts were incubated with the secondary antibody (Alexa-Fluor-488-labeled goat anti-mouse IgG) for 1 h at room temperature. After washing three times in blocking solution, the embryos and blastocysts were mounted on glass slides with Vectashield containing DAPI. Slides were examined under epifluorescence using an LSM 700 confocal microscope (Zeiss, Jena, Germany). The fluorescence levels were analyzed using ImageJ software.

### 2.8. TUNEL Assay

Terminal deoxynucleotidyl transferase-mediated dUTP-digoxigenin nick end-labeling (TUNEL) assay was carried out as described previously with some modifications [30]. Apoptotic cells in blastocysts were detected by using an In Situ Cell Death Detection kit (Roche, Basel, Switzerland). Fixed blastocysts were washed three times in PBS/PVA and permeabilized with 1% Triton X-100 in PBS for 1 h at room temperature. After washing three times with PBS/PVA, the blastocysts were incubated with fluorescein-conjugated dUTP and terminal deoxynucleotidyl transferase at 38.5 °C for 1 h. After washing three times with PBS/PVA, the blastocysts were mounted on glass slides with mounting solution containing 1.5 µg/mL DAPI. TUNEL- and DAPI-stained nuclei were captured using a fluorescence microscope (Olympus).

### 2.9. Real-Time Quantitative PCR (qPCR)

Gene expression analysis using qPCR was carried out as described previously with some modifications [31]. Poly(A) mRNAs were extracted from twenty Day-2 embryos and five Day-6 blastocysts using a Dynabeads mRNA Direct kit (Invitrogen). After thawing, the samples were lysed in 200 µL of lysis buffer at room temperature for 10 min. Subsequently, 10 µL of Dynabeads oligo(dT) 25 was added to each sample. The samples were incubated for 5 min at room temperature. The beads were hybridized for 5 min and then separated from the binding buffer using a Dynal magnetic bar (Invitrogen). Poly(A) mRNAs bound to the beads were washed three times in buffers A and B individually. Then, 15 µL Tris-HCl buffer was added to separate poly(A) mRNAs. The poly(A) mRNAs were then reverse-transcribed using a PrimeScript™ RT Reagent kit with gDNA Eraser (Takara Bio Inc., Shiga, Japan) according to the manufacturer’s protocol. The reverse-transcription reaction was carried out at 37 °C for 15 min. The termination reaction was performed at 85 °C for 5 min. The synthesized cDNA samples were used as templates for qPCR analysis. Subsequently, qPCR was performed using SYBR Premix Ex Tap (TaKaRa, Shiga, Japan) with the MX3000P QPCR system (Agilent, Santa Clara, CA, USA) under the following conditions: Initial denaturation at 95 °C for 10 min; 40 cycles of denaturation (30 s at 95 °C), annealing (30 s at 60 °C), and extension (30 s at 72 °C); a final extension at 72 °C for 5 min. For comparative analyses, glyceraldehyde-3 phosphate dehydrogenase (*GAPDH*) was used as the internal standard for each group. Primer sequences are listed in Table 1.

### 2.10. Experimental Design

In experiment 1, a total of 684 embryos was used in five independent replicates. To compare the effects of various concentrations of lycopene on in vitro development of IVF embryos, IVF embryos were cultured in IVC medium supplemented with 0, 0.02, 0.05, 0.1, or 0.2 μM lycopene during the entire period of IVC. We then evaluated the developmental competence of groups treated with various concentrations of lycopene, using parameters such as the inner cell mass (ICM) to trophectoderm (TE) ratio, total cell number, and rate of blastocyst formation. In experiment 2, a total of 84 Day-2 embryos and 72 Day-6 blastocysts was used in three independent replicates. To assess the antioxidative effect of lycopene on IVF embryos, we investigated intracellular ROS levels in Day-2 embryos and Day-6 blastocysts treated with or without an optimal concentration of lycopene. In experiment 3, a total of 104 Day-2 embryos and 88 Day-6 blastocysts was used in three independent replicates. To investigate the effect of lycopene on mitochondrial membrane potential in IVF embryos, we performed JC-1 analysis and evaluated the ratio of red fluorescence (J-aggregate, high membrane potential) to green fluorescence (J-monomer, low membrane potential) of Day-2 embryos and Day-6 blastocysts treated with or without an optimal concentration of lycopene. In experiment 4, a total of 76 Day-2 embryos and 52 Day-6 blastocysts was used in three independent replicates. To investigate changes in the expression levels of cytochrome c in IVF embryos, we evaluated cytochrome c expression based on the immunofluorescence of Day-2 embryos and Day-6 blastocysts treated with or without an optimal concentration of lycopene. In experiment 5, a total of 26 and 16 Day-6 blastocysts was used in three independent replicates to investigate changes in expression of cleaved caspase-3 and apoptosis in IVF blastocysts, respectively. We evaluated the number and percentage of cleaved caspase-3 and TUNEL positive cells in Day-6 blastocysts. In experiment 6, a total of 120 Day-2 embryos and 30 Day-6 blastocysts was used in at least five independent replicates. We investigated the expression levels of mRNAs related to antioxidant enzymes and apoptosis in Day-2 embryos and Day-6 blastocysts.

### 2.11. Statistical Analysis

SigmaStat statistical program (SPSS, Inc., Chicago, IL, USA) was used for all statistical analysis. All experiments were repeated at least three times. For comparisons of three or more groups, one-way analysis of variance was performed, followed by Tukey’s post hoc test. The scores of the two groups were compared using Student’s *t*-test. All results are expressed as the mean ± SEM. A value of *p* < 0.05 was considered to denote statistical significance.

## 3. Results

### 3.1. Effects of Various Concentrations of Lycopene on In Vitro Development of IVF Embryos

**Figure 1:**
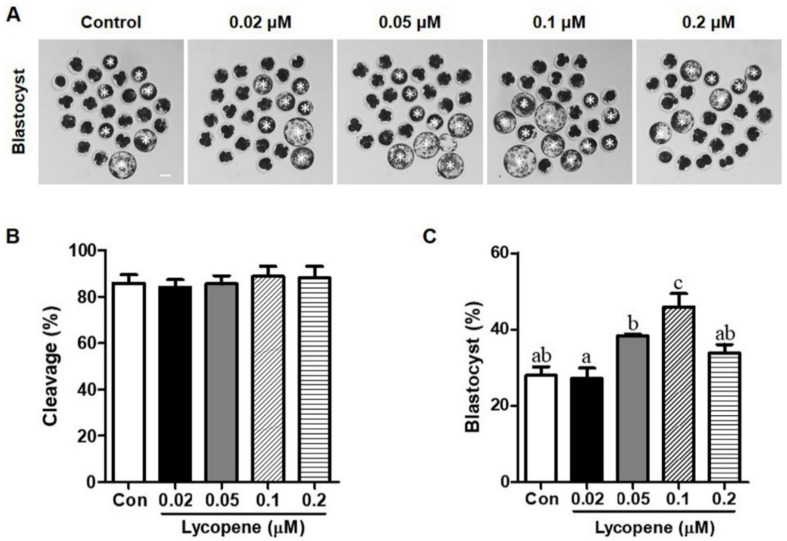
The effect of various concentrations of lycopene during the entire period of in vitro culture on the development of porcine IVF embryos. (**A**) Representative images of Day-6 IVF embryos and (**B**) rates of cleavage and (**C**) blastocyst formation in embryos supplemented with 0, 0.02, 0.05, 0.1, and 0.2 μM lycopene. Different superscript letters (a–c) represent the significant difference (*p* < 0.05). Bar = 100 μM. Abbreviations: IVF, in vitro fertilized; Con, control.

**Figure 2:**
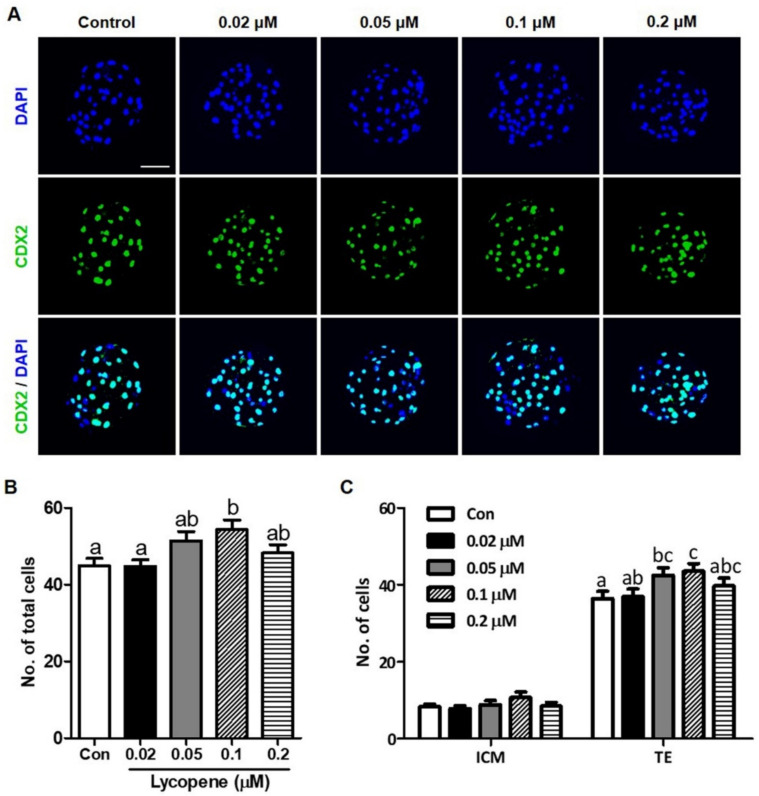
Effect of lycopene on ICM, TE ratio, and total cell number of porcine IVF embryos. (**A**) Representative images of differential staining of Day-6 IVF blastocysts treated with various concentrations (0.02, 0.05, 0.1, and 0.2 μM) of lycopene. (**B**) Total cell number and (**C**) ICM and TE ratio of blastocysts in the indicated groups. Different superscript letters (a–c) represent the significant difference (*p* < 0.05). Bar = 100 μM. Abbreviations: ICM, inner cell mass; TE, trophectoderm; IVF, in vitro fertilized; DAPI, 4′,6-diamidino-2-phenylindole; CDX2, caudal type homeobox 2; Con, control.

To examine the effects of lycopene on the in vitro development of preimplantation porcine embryos, IVF embryos were treated with 0 (control), 0.02, 0.05, 0.1, or 0.2 µM lycopene for the entire period of IVC. Figure 1 shows that although there were no differences in cleavage rates between the lycopene-treated groups and the control group, blastocyst formation was significantly increased with the addition of 0.1 µM lycopene (45.9 ± 3.5). However, no difference in blastocyst formation was observed in the 0.02, 0.05, and 0.2 µM lycopene-treated groups (27.1 ± 2.7, 38.3 ± 0.5, and 34.6 ± 1.7, respectively) compared to the control group (27.9 ± 2.2). Figure 2 shows that the TE cell number was significantly increased with the addition of 0.1 µM lycopene (43.6 ± 1.9) compared to the control group (36.5 ± 1.8). However, there was no difference in ICM cell number between the group treated with 0.1 µM lycopene (10.7 ± 1.4) and the control group (8.4 ± 0.6). As a result, the total cell number was significantly increased in the group treated with 0.1 µM lycopene (54.3 ± 2.5) compared to the control group (44.8 ± 1.9).

### 3.2. Effect of Lycopene on Intracellular ROS Levels in IVF Embryos

**Figure 3:**
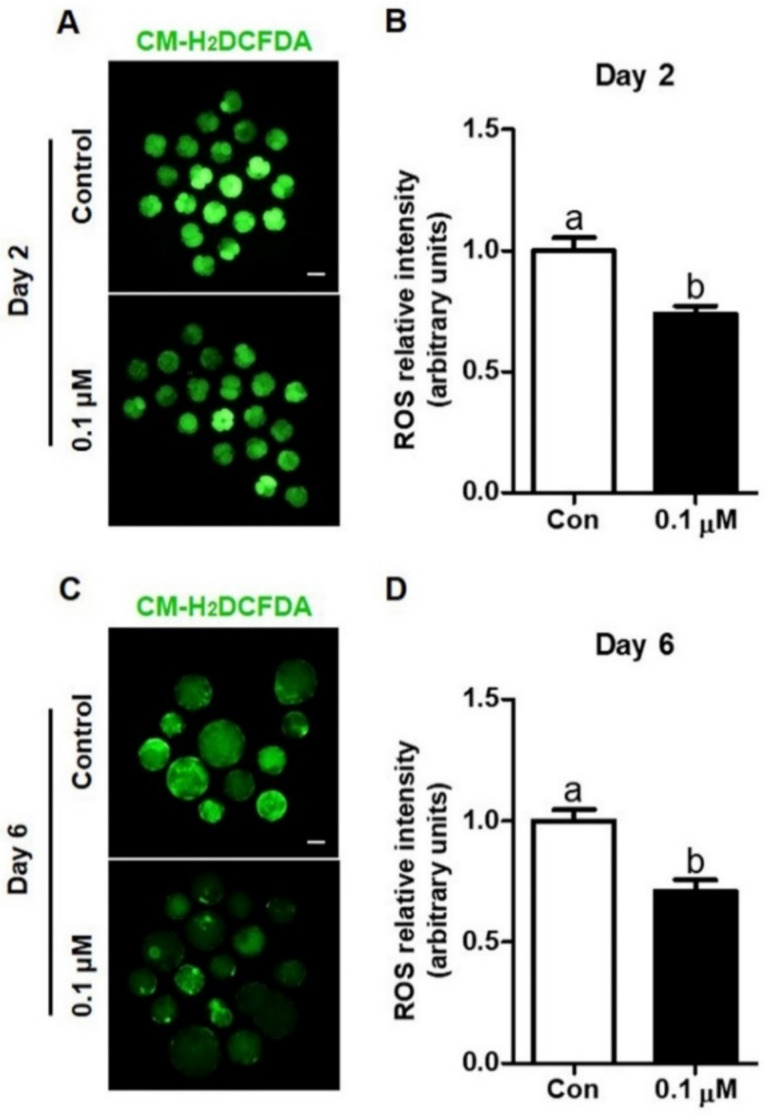
Effect of lycopene on the intracellular ROS levels in porcine IVF embryos. (**A**,**C**) Representative images of CM-H_2_DCFDA staining of Day-2 embryos and Day-6 blastocysts treated with or without 0.1 μM lycopene. (**B**,**D**) Intracellular ROS levels in Day-2 embryos and Day-6 blastocysts in the indicated groups. Different superscript letters (a,b) represent the significant difference (*p* < 0.05). Bar = 100 μM. Abbreviations: ROS, reactive oxygen species; IVF, in vitro fertilized; CM-H_2_DCFDA, 5-[and-6]-chloromethyl-20, 70-dichlorodihydro-fluorescein diacetate, acetyl ester; Con, control; 0.1 μM, 0.1 μM lycopene.

To investigate the antioxidative effect of lycopene in IVF porcine embryos, intracellular ROS levels were analyzed in IVF embryos cultured with IVC medium supplemented with 0 (control) or 0.1 µM lycopene. Figure 3 shows that intracellular ROS levels in Day-2 embryos and Day-6 blastocysts treated with 0.1 µM lycopene were significantly lower than their corresponding control groups.

### 3.3. Effect of Lycopene on Mitochondrial Membrane Potential in IVF Embryos

**Figure 4:**
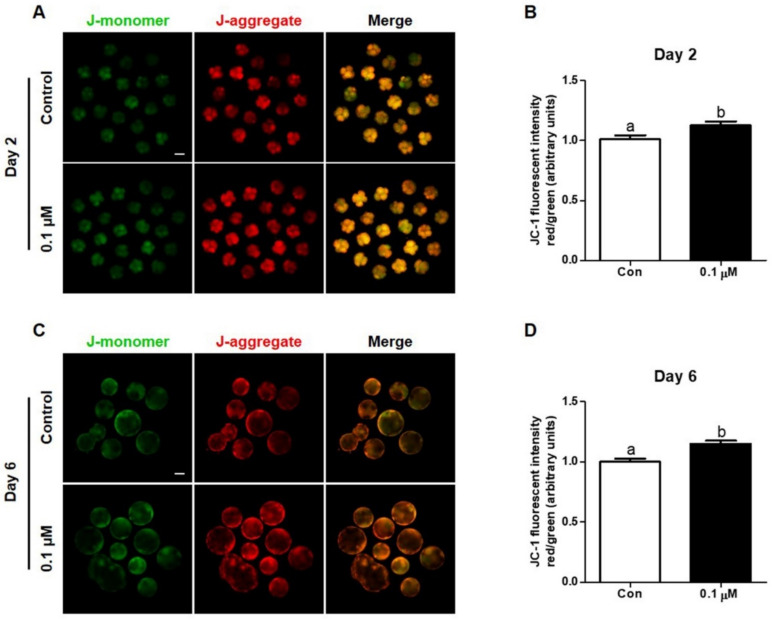
Effect of lycopene on mitochondria function in porcine IVF embryos. (**A**,**C**) Representative images of JC-1 staining of Day-2 embryos and Day-6 blastocysts treated with or without 0.1 μM lycopene. (**B**,**D**) Quantification of the ratio of fluorescence intensity (red/green) in Day-2 embryos and Day-6 blastocysts in the indicated groups. Different superscript letters (a,b) represent the significant difference (*p* < 0.05). Bar = 100 μM. Abbreviations: IVF, in vitro fertilized; Con, control; 0.1 μM, 0.1 μM lycopene.

To investigate the effect of lycopene on mitochondrial membrane potential in IVF embryos, Day-2 embryos and Day-6 blastocysts from the control and 0.1 μM lycopene-treated groups were used for the JC-1 assay. Figure 4 shows that the mitochondrial membrane potential of Day-2 embryos and Day-6 blastocysts treated with 0.1 μM lycopene was significantly higher than levels in their corresponding control groups.

### 3.4. Effect of Lycopene on Cytochrome C Expression in IVF Embryos

**Figure 5:**
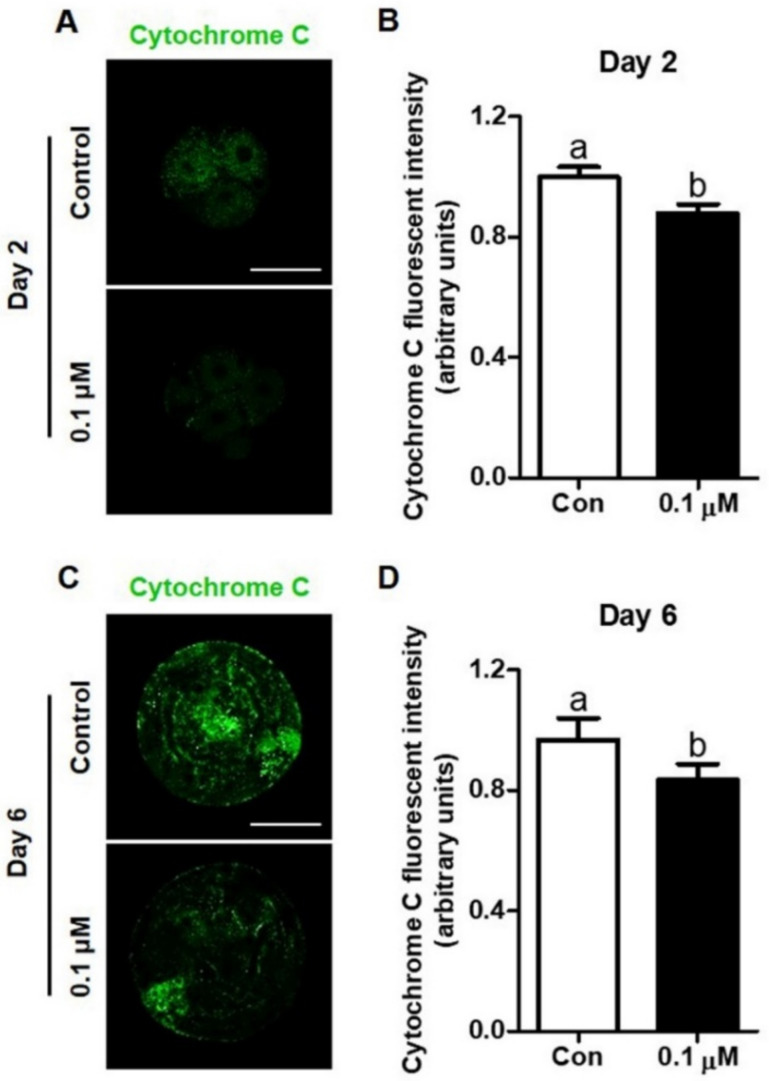
Effect of lycopene on the expression of cytochrome c in porcine IVF embryos. (**A**,**C**) Representative images of Day-2 embryos and Day-6 blastocysts of the indicated groups stained with an antibody directed against cytochrome c and (**B**,**D**) comparison of expression in Day-2 embryos and Day-6 blastocysts in the indicated groups. Different superscript letters (a,b) represent the significant difference (*p* < 0.05). Bar = 100 μM. Abbreviations: IVF, in vitro fertilized; Con, control; 0.1 μM, 0.1 μM lycopene.

The expression of cytochrome c in Day-2 embryos and Day-6 blastocysts treated with 0.1 μM lycopene was analyzed using immunocytochemical staining. Based on the results, Day-2 embryos and Day-6 blastocysts from the 0.1 µM lycopene-treated group exhibited significantly lower expression of cytochrome c, compared to their corresponding control groups (Figure 5).

### 3.5. Effect of Lycopene on the Expression of Cleaved Caspase-3 and Apoptosis Levels in IVF Embryos

**Figure 6:**
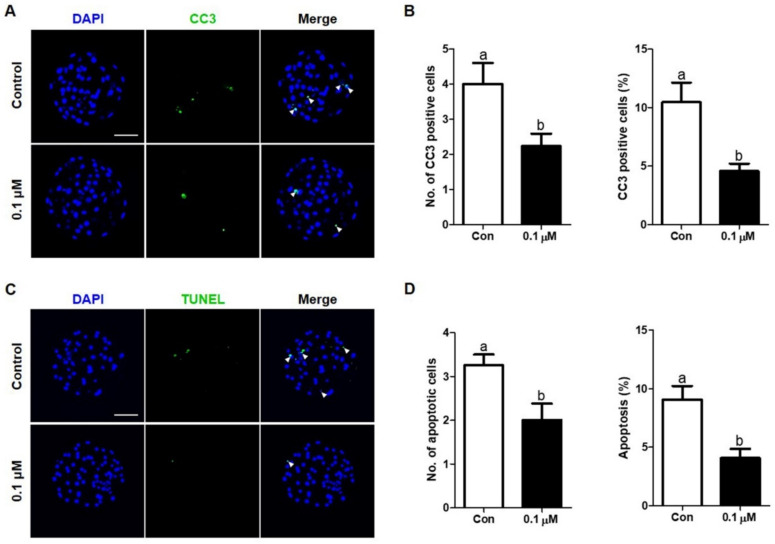
Effect of lycopene on CC3 expression and apoptosis levels in porcine IVF blastocysts. (**A**) Representative images of Day-6 IVF blastocysts of the indicated groups stained with an antibody directed against cleaved caspase 3 and (**B**) comparison of number and percentage of CC3-positive cells in the indicated groups. (**C**) Representative images of TUNEL staining of Day-6 IVF blastocysts treated with or without 0.1 μM lycopene and (**D**) comparison of number and percentage of TUNEL-positive cells in the indicated groups. Different superscript letters (a,b) represent the significant difference (*p* < 0.05). Bar = 100 μM. Abbreviations: CC3, cleaved caspase 3; IVF, in vitro fertilized; TUNEL, terminal deoxynucleotidyl transferase-mediated dUTP-digoxigenin nick end-labeling; Con, control; 0.1 μM, 0.1 μM lycopene.

The expression of cleaved caspase-3 in Day-6 blastocysts treated with 0.1 μM lycopene was examined using immunocytochemical staining. The number and percentage of cleaved caspase-3 in Day-6 blastocysts was significantly decreased in the 0.1 µM lycopene-treated group compared to the control group (Figure 6A,B). We also investigated the presence of apoptotic cells in Day-6 blastocysts using the TUNEL assay (Figure 6C,D). As shown in Figure 6, the number and percentage of apoptotic cells in Day-6 blastocysts were significantly lower in the 0.1 µM lycopene-treated group compared to the control group.

### 3.6. Effect of Lycopene on the Expression of Antioxidant Enzyme- and Apoptosis-Related mRNAs in IVF Embryos

**Figure 7:**
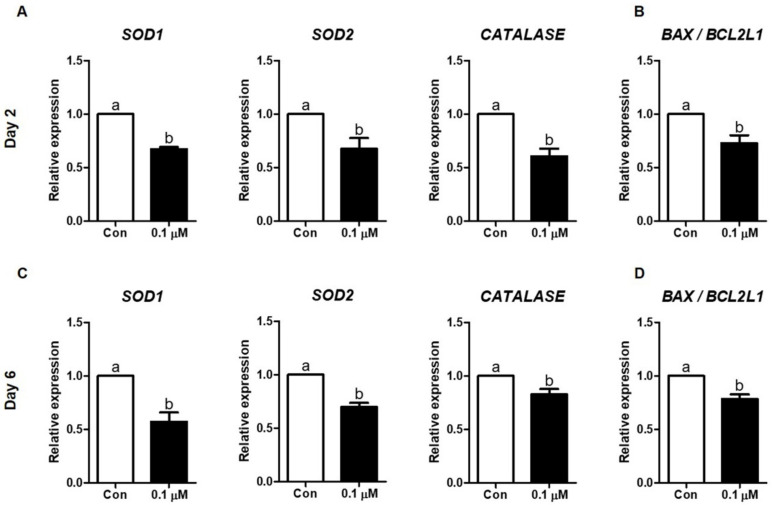
Effect of lycopene on expression levels of antioxidant enzymes- and apoptosis-related mRNAs in porcine IVF embryos. (**A**,**C**) Antioxidant enzymes-related mRNAs in Day-2 and Day-6 IVF embryos. (**B**,**D**) Apoptosis-related mRNAs in Day-2 and Day-6 IVF embryos. Different superscript letters (a,b) represent the significant difference (*p* < 0.05). Abbreviations: IVF, in vitro fertilized; Con, control; 0.1 μM, 0.1 μM lycopene.

The expression of antioxidant enzyme and apoptosis mRNAs in IVF embryos cultured with or without 0.1 µM lycopene was investigated using qPCR. The relative mRNA levels of *SOD1*, *SOD2*, and *CATALASE* (antioxidant enzyme genes) were significantly lower in the 0.1 µM lycopene-treated group compared to the control group on Day 2 and Day In addition, the *BAX/BCL2L1* ratio (apoptosis genes) was significantly decreased in the 0.1 µM lycopene-treated group compared to the control group (Figure 7).

## 4. Discussion

The present study demonstrated that lycopene supplementation during IVC improved developmental competence and reduced mitochondria-dependent apoptosis in porcine IVF embryos. Lycopene prevented intracellular ROS production, improved mitochondrial membrane potential, decreased cytochrome c release and caspase 3 activation, and consequently led to reduced apoptosis. These results suggest that lycopene enhanced the developmental competence of porcine IVF embryos by regulating mitochondria-dependent apoptosis induced by oxidative stress in vitro.

The production of high-quality embryos enhances the establishment of efficient animal models for biomedical research. However, in contrast to the interior of the oviduct, the in vitro environment exposes embryos to various stressful conditions, leading to disproportionate amounts of antioxidants and free radicals in the cytoplasm of the embryos [32]. Therefore, reducing oxidative stress is important to enhance the developmental competence and quality of IVP porcine embryos. In this study, we investigated the effects of lycopene on the developmental competence of porcine IVF embryos and the mechanisms involved in mitochondria-dependent apoptotic pathways. First, we evaluated the effects of various concentrations of lycopene (0, 0.02, 0.05, 0.1, and 0.2 μM) on the preimplantation development of IVF embryos. Our findings indicated that 0.1 μM lycopene supplementation during the entire period of IVC significantly increased the rate of blastocyst formation and the total cell numbers on Day 6, suggesting that 0.1 μM lycopene is the optimal concentration for embryonic development. This concentration is similar to human serum and follicular lycopene concentrations based on previous studies, which reported that lycopene concentration in human serum is 0.15 ± 0.14 μmol/L and that in follicular fluid is 0.06 ± 0.02 μmol/L [33,34].

In cellular metabolism, ROS are generated by energy-producing pathways during mitochondrial respiration [35]. Although biological oxidation often produces unwanted ROS, low levels of ROS are important for physiological signal transduction pathways in the embryo [36]. However, excessive generation of ROS induced by in vitro environmental stress damages cell structures and activates apoptotic pathways in many different types of cells [37]. In addition, excessive levels of intracellular ROS caused developmental arrest and apoptosis during IVC of bovine embryos [38]. To prevent ROS-induced damage of embryos, many studies have used antioxidants such as vitamin C [16], glutathione [39], and fetal bovine serum [40] to enhance developmental competence by scavenging ROS. In this study, 0.1 μM lycopene significantly decreased intracellular ROS levels in porcine IVF embryos. It also significantly decreased the number and percentage of apoptotic cells in blastocysts compared to the control group. Therefore, the results of the present study clearly demonstrate that the use of lycopene, as an ROS scavenger in the culture medium of IVF embryos, prevented not only the production of ROS but also cellular apoptosis.

An increase in cellular ROS levels can cause mitochondrial dysfunction. Mitochondria are important for regulating energy production and function as storage organelles for calcium ions [41]. In addition, mitochondria are closely associated with cell differentiation, cellular apoptosis pathways, and cell viability [42]. Furthermore, the number of mitochondria in the cytoplasm has an effect on embryonic development [43]. Previous studies have indicated that the mitochondrial membrane potential, a key marker of the quality of embryos, can be used as an indicator to evaluate the developmental competence of preimplantation embryos in pigs [44,45]. The dissipation of mitochondrial membrane potential has been shown to cause the release of cytochrome c, which is one of the messengers of mitochondria-dependent apoptotic pathways [46]. The release of cytochrome c triggers the activation of caspases, which eventually results in apoptosis [47]. In this study, treatment with 0.1 μM lycopene significantly increased the mitochondrial membrane potential in IVF embryos compared with the control group on Day 2 and Day 6, indicating that lycopene supplementation during IVC improved mitochondrial function. These results are consistent with those of a previous study that demonstrated that lycopene restored the mitochondrial membrane potential and decreased ROS generation in damaged primary mouse neurons [48]. In addition, to determine whether apoptotic events in blastocysts were induced by the release of cytochrome c and the activation of pro-caspase 3 protein, we evaluated the expression of cytochrome c and cleaved caspase-3 using immunofluorescence. The results clearly indicated that the expression of cytochrome c and cleaved caspase 3 decreased upon treatment with lycopene. This suggests that lycopene decreases the apoptosis levels in blastocysts by reducing mitochondria-dependent apoptosis.

Superoxide anion (O_2_^−^) generated by oxidative metabolism in mitochondria is converted by SOD to hydrogen peroxide (H_2_O_2_) [49], which is then broken down into water and oxygen by catalase [50]. In a previous study, it was shown that low levels of intracellular ROS can reduce the mRNA levels of *SOD1*, *SOD2*, and *CATALASE* [51]. In addition, the downregulation of *SOD* mRNA transcripts is related to greater embryonic developmental competence [52]. These results are consistent with those of the current study, which showed that lycopene treatment significantly reduced *SOD1*, *SOD2*, and *CATALASE* mRNA expression in Day-2 embryos and Day-6 blastocysts compared to the control group. This implies that the presence of lycopene in the IVC medium may increase extrinsic antioxidant protection, which possibly decreases the synthesis of endogenous antioxidant enzymes such as SOD1, SOD2, and catalase in embryos to basal levels to retain the redox equilibrium.

Mammalian embryos such as mouse, bovine, and porcine embryos have been studied as models for understanding biochemical and physiological regulations of human embryos in general. They can be proposed as tools to determine the applicability of the methods, techniques, and materials used in human embryo culture. Therefore, our results showing the beneficial effects of lycopene on embryonic development in pigs suggest the applicability of lycopene in fertility treatment in humans.

## 5. Conclusions

In conclusion, treatment with 0.1 μM lycopene significantly increased the blastocyst formation rate and total cell numbers during early porcine IVF embryo development. In addition, lycopene prevented ROS production, improved mitochondrial function, and decreased cytochrome c expression in IVF embryos. Subsequently, cleaved caspase-3 activity and apoptosis in the blastocysts were attenuated by lycopene treatment during IVC. Taken together, these results indicate that lycopene exerts beneficial effects on the development of porcine IVF embryos by preventing oxidative stress, mitochondrial dysfunction, and subsequent apoptosis. These findings suggest the potential contribution of lycopene supplementation during embryo culture to efficient pig production. Moreover, this investigation provides novel insights that highlight the beneficial effects of lycopene on embryo culture in a porcine model that may be applicable to fertility treatment in humans.

## Figures and Tables

**Table 1:** PCR sequences used for real-time quantitative PCR (qPCR).

Genes	Primer Sequence (5′-3′)	Accession Number
*GAPDH*	F-CCCTGAGACACGATGGTGAA	NM_001206359.1
	R-GGAGGTCAATGAAGGGGTCA	
*SOD1*	F-GGTGGGCCAAAGGATCAAGA	NM_001190422.1
	R-TACACAGTGGCCACACCATC	
*SOD2*	F-GGTGGAGGCCACATCAATCA	NM_214127.2
	R-AACAAGGGCAATCTGCAAG	
*CATALASE*	F-TGTACCCGCTATTCTGGGGA	NM_214301.2
	R-TCACACAGGCGTTTCCTCTC	
*BAX*	F-CGATCTCGAAGGAAGTCCAG	XM_003127290.5
	R-AAGCGCATTGGAGATGAACT	
*BCL2L1*	F-AGGGCATTCAGTGACCTGAC	NM_214285.1
	R-CGATCCGACTCACCAATACC	

F: forward, R: reverse.

## Data Availability

Data is contained within the article.
